# Electrosurgery and Temperature Increase in Tissue With a Passive Metal Implant

**DOI:** 10.3389/fsurg.2019.00008

**Published:** 2019-03-12

**Authors:** Tormod Martinsen, Fred Johan Pettersen, Håvard Kalvøy, Christian Tronstad, Gunnvald Kvarstein, Andre Bakken, Jan Olav Høgetveit, Ørjan G. Martinsen, Sverre Grimnes, Lars Frich

**Affiliations:** ^1^Department of Clinical and Biomedical Engineering, Oslo University Hospital Rikshospitalet, Oslo, Norway; ^2^Department of Clinical Medicine, UiT, The Arctic University of Norway, Tromsø, Norway; ^3^Department of Physics, University of Oslo, Oslo, Norway; ^4^Department of Plastic and Reconstructive Surgery, Oslo University Hospital Radiumhospitalet, Oslo, Norway

**Keywords:** electrosurgery, metal-implant, temperature-increase, safety, burns, tissue-damage, tissue-temperature, optical measurement

## Abstract

**Importance:** During monopolar electrosurgery in patients, current paths can be influenced by metal implants, which can cause unintentional tissue heating in proximity to implants. Guidelines concerning electrosurgery and active implants such as pacemakers or implantable cardioverter defibrillators have been published, but most describe interference between electrosurgery and the active implant rather than the risk of unintended tissue heating. Tissue heating in proximity to implants during electrosurgery may cause an increased risk of patient injury.

**Objective:** To determine the temperature of tissue close to metal implants during electrosurgery in an *in-vitro* model.

**Design, Setting, and Participants:** Thirty tissue samples (15 with a metal implant placed in center, 15 controls without implant) were placed in an *in vitro* measurement chamber. Electrosurgery was applied at 5–60 W with the active electrode at three defined distances from the implant while temperatures at four defined distances from the implant were measured using fiber-optic sensors.

**Main Outcomes and Measures:** Tissue temperature increase at the four tissue sites was determined for all power levels and each of the electrode-to-implant distances. Based on a linear mixed effects model analysis, the primary outcomes were the difference in temperature increase between implant and control tissue, and the estimated temperature increase per watt per minute.

**Results:** Tissues with an implant had higher temperature increases than controls at all power levels after 1 min of applied electrosurgery (mean difference of 0.16°C at 5 W, 0.50°C at 15 W, 1.11°C at 30 W, and 2.22°C at 60 W, all with *p* < 0.001). Temperature increase close to the implant was estimated to be 0.088°C/W/min (95% CI: 0.078–0.099°C/W/min; *p* < 0.001). Temperature could increase to above 43°C after 1 min of 60 W. Active electrode position had no significant effect on temperature increases for tissues with implant (*p* = 0.6).

**Conclusions and Relevance:** The temperature of tissue close to a metal implant increases with passing electrosurgery current. There is a significant risk of high tissue temperature when long activation times or high power levels are used.

## Introduction

Electrosurgery involves the application of an ~500 kHz, high-frequency, electrical current to biological tissue as a means to cut, coagulate, desiccate, or fulgurate that tissue. During bipolar electrosurgery, the current is applied locally (i.e., between two tips of a forceps), with no need for a dispersive electrode. During monopolar electrosurgery, the current passes from the active electrode through the tissue to a dispersive electrode. During electrosurgery, high current density in close proximity to the active electrode causes temperature increases that achieve the desired outcome for the tissue (1). Electrical current distribution in tissue has been discussed since electrosurgery was introduced in ~1920 (2, 3). It is generally accepted that the electrical current path between the active electrode and the dispersive electrode in monopolar electrosurgery can be influenced by a metal implant. However, how a highly conductive implant actually changes the current density in the tissue is not properly documented in the current literature. This lack of knowledge can lead to uncertainty regarding surgery and preparation for surgery, thereby affecting patient safety (4–9). Electrosurgery is frequently associated with reported unwanted events and is listed in the Emergency Care Research Institute's (ECRI) list of top 10 reported patient safety events for 2012 (4).

There are certain safety challenges regarding the use of electrosurgery. Sparks from electrosurgery can cause a surgical fire in the operating room. The risk is increased if the tissue is saturated with high oxygen levels or in combination with flammable liquids like alcohol (4, 10–12). Capacitive coupling can transfer energy from the high-frequency electrosurgery current to its surroundings (the antenna effect). This is typical when wires or other conductive parts of other devices in contact with the patient are placed close to the electrosurgical active electrode and electrode wire (1, 6, 12–15). Burns of the tissue surrounding the active electrode (16, 17) or burns caused by the active electrode can occur (13, 17). Burns can also be caused by electrode insulation failure (13). In addition, burns associated with the dispersive electrode with insufficient contact area, poor placement, or spilled fluid have been reported (12, 14). Electrosurgery can disturb or even alter the functionality of active implants like pacemakers, implantable cardioverter defibrillators, deep brain stimulators (DBS), or cochlear implants due to electromagnetic interference (9, 12, 15, 18–28). Furthermore, tissue burns can develop in certain areas around electrical conducting implants (active or passive). The highest risk typically occurs in small exposed areas in electrical contact with the tissue (9, 12, 19–21, 23–27, 29–31).

Interference between electrosurgery energy and the active implant have been thoroughly discussed in the literature; however, few studies have investigated burn hazards caused by electric conduction through implants, both active and passive (1, 9, 12, 15, 18, 20, 21, 23–27, 29, 32). There is no doubt that this sometimes influences the situation in the OR. Therefore, we aimed to contribute more knowledge about the mechanisms of currents in tissues with implants.

Some of the variables that can influence the current path around an implant and the temperature of the tissue are as follows: electrosurgery power; duration of activation; time of non-activation (pause); the amount of electrical contact area between the active electrode and tissue (contact impedance); how the current is directed in the active electrosurgery area; where the implant is located and placement of the dispersive electrode; the shape of the implant, including sharpness of the ends; implant length; implant electrical characteristics; implant size, and thermal capacity; whether the implant has isolated areas that end in a small area (e.g., a stimulation electrode); tissue electrical characteristics; and tissue vascularization.

This study aimed to investigate the relationship between the temperature of tissue close to a passive metal implant and electrical power of the electrosurgery unit. By this we studied if the electrosurgery current will follow the lowest electrical impedance path and be channelized through the implant. We also studied whether the distance between an active electrode and a metal implant influences the tissue temperature close to the implant. This was accomplished by testing the following three hypotheses (H1, H2, and H3): H1, there is a local temperature increase in the tissue close to a metal implant with passing monopolar electrosurgery current; H2, the temperature increase in the tissue close to a metal implant with passing monopolar electrosurgery is influenced by different applied power levels; and H3, the temperature increase in the tissue close to a metal implant with passing monopolar electrosurgery is influenced by the distance between the active electrode and implant.

## Methods

To study the effects of an implant on local tissue temperature increase, a factorial design was used (Table 1). Four levels of applied electrical power typically used during various types of surgery were used (5 W, 15 W, 30 W, 60 W). To observe the possible effect of successive power on tissue temperature change, power was applied in increasing steps and then in decreasing steps. This was performed to test for hysteresis between tissue heating and applied energy. To study the temperature change as a function of the distance between the implant and the active electrode, three positions (close, 27.8 mm; middle, 47.8 mm; far, 67.8 mm) were used for the active electrode. Four different positions in the vicinity of the implant were used for determining the heating effect based on the increase in temperature after 1 min of applied electrosurgical current. Thirty tissue samples were measured in a custom-made, temperature-regulated measurement chamber.

**Table 1:** Overview of factors and levels used in the experimental design of the study.

**Factor**	**Level 1**	**Level 2**	**Level 3**	**Level 4**
Implant	With	Without		
Power	5 W	15 W	30 W	60 W
Position	Close (27.8 mm)	Middle (47.8 mm)	Far (67.8 mm)	
Power step	Increasing	Decreasing (Hysteresis test)		

*The power step factor represents the direction of steps in applied power, which was first increasing and then decreasing to the previous levels in order to test for possible hysteresis effects*.

**Figure 1:**
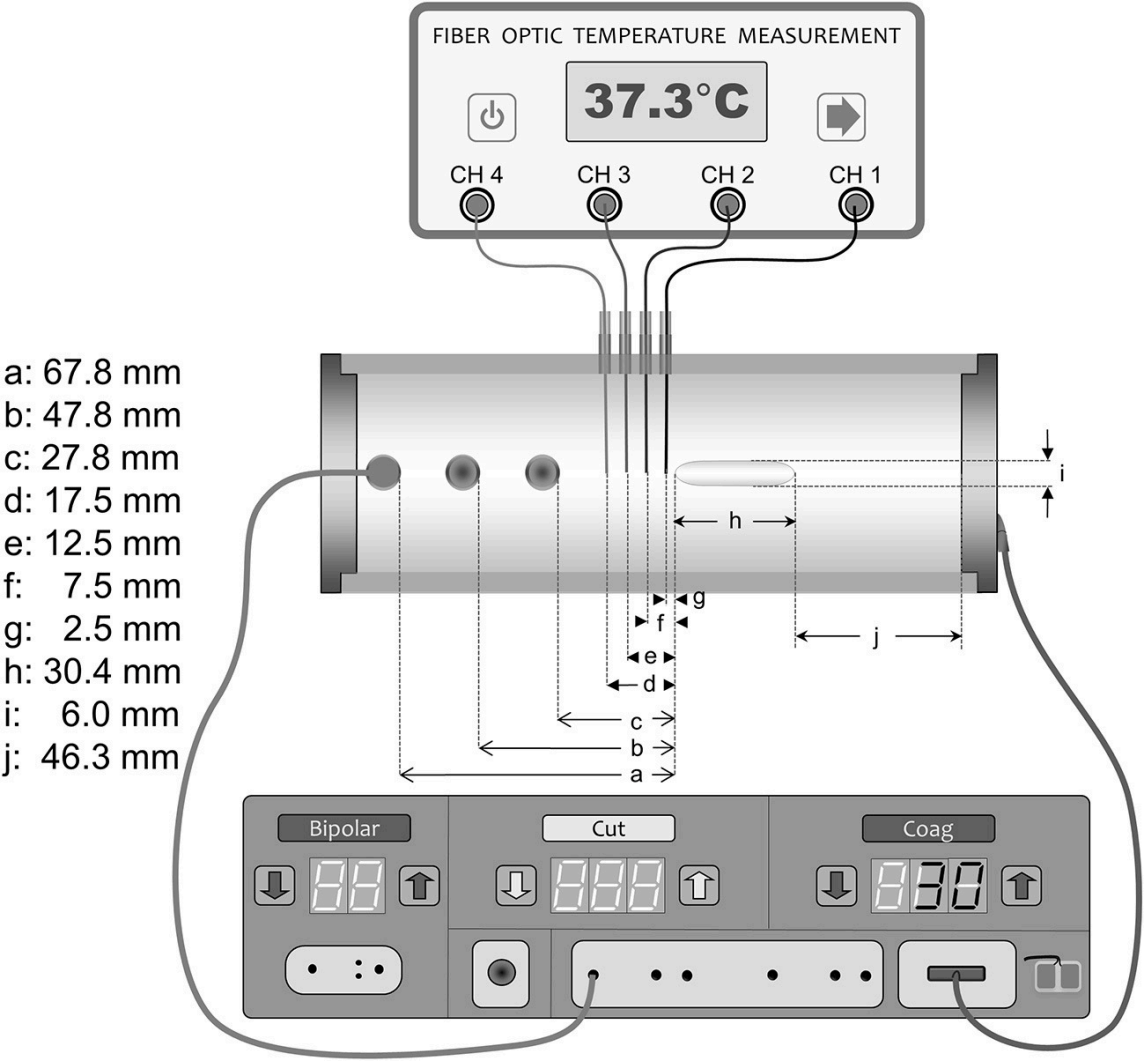
Measurement chamber and setup.

The measurement chamber was created using a cylindrical acrylic tube (Figure 1). The chamber had holes on each side for horizontal mounting of the active electrode rod in three different positions.

**Figure 2:**
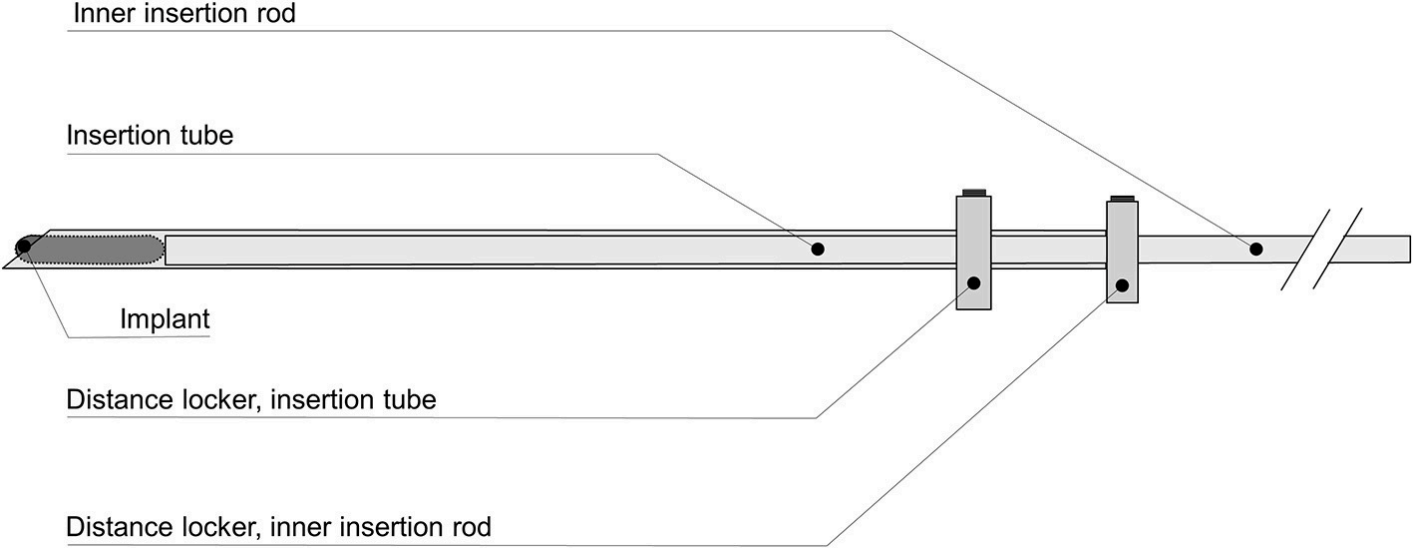
Insertion tube.

**Figure 3:**
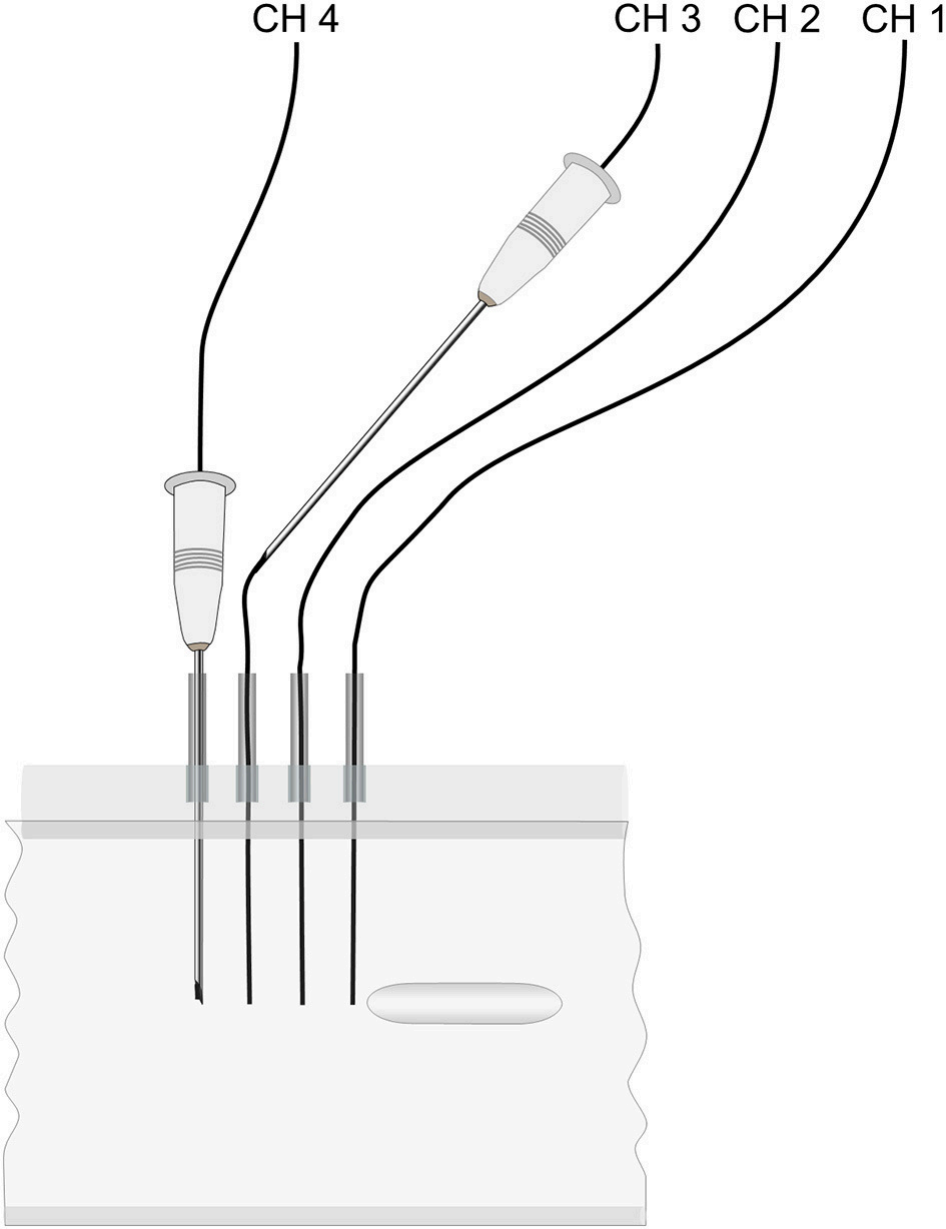
Sensor placement guides.

Fresh porcine muscle tissue samples of ~690 g were punched out with a punch tool made from a cylinder with an inner radius of 54 mm. The active electrode consisted of a round stainless-steel rod 9 mm in diameter and with a trocar-shaped end. A stainless-steel disc with a radius of 45 mm was mounted as the dispersive electrode at the other end of the chamber. The *in vitro* chamber containing tissue and the active electrode was placed in an incubator chamber (Microplate Incubator; IPS Diagnostics, Shanghai, China). The chamber was modified with a new cover measuring 90 × 250 × 305 mm. The cover had a 5-mm slit on the top according to the temperature sensor insertion guides. To provide a stable temperature environment around the measuring chamber, the incubator was modified with a 2.3-kg massive aluminum block. A SAE 304 (A2) stainless-steel implant (30.4 × 6.0 mm) shaped like a round rod was inserted in the tissue (Figure 1). The implant was designed with an elliptic shape on both ends to provide a smooth electrosurgery current entrance to the implant. Precise placement of the implant according to the position of the temperature sensors in the middle of the model was performed with an insertion tube (Figure 2). The chamber had four insertion holes for non-conductive fiber-optic temperature sensors (CH1–CH4) (Figures 1, 3). During this study, we used isolated, non-conductive, fiber-optic temperature sensors to avoid the “antenna effect” (3, 6). Temperature measurements were performed with four fiber-optic sensors (OTG-MPK5-10-62F2.5-1.5PTFE-XN-10PIT-M2; Opsens, Quebec, Canada). The measurement areas of the temperature sensors in the tips were placed 2.5, 7.5, 12.5, and 17.5 mm in front of the implant along the long axis in the center of the measurement chamber. Precise placement of the fiber-optic temperature sensors was achieved with 14-G syringe needles (2 × 80-mm) (Figure 3). The four fiber-optic temperature sensors were then connected to the four-channel fiber-optic measuring device (TMS-G4-10-100ST-M2; Opsens). The unit was connected to a computer to log the temperatures with Opsens software. The four temperature measurement positions were used to determine the distribution of the temperature increase associated with the presence of the implant in combination with electrosurgery current. Therefore, we could determine where the temperature was highest during activation of the electrosurgery unit. To analyze the influence of the implant, the sensor with the highest temperature increases was selected (CH1, closest to the implant). This study was performed with the Valleylab Force-fx electrosurgery unit (Covidien/Medtronic, Galway, Ireland). A 4-mm banana male connector with a cable was connected to the active output on the electrosurgery unit. The other side of the cable was connected to the active electrode. The dispersive electrode was connected using a modified dispersive cable.

Measurements were performed in 15 tissues with implants and 15 without implants which were used as a control group. All measurements were performed using the electrosurgery unit in the coagulation fulgurate mode. Each single measurement sequence was performed using 5-, 15-, 30-, and 60-W power steps of the electrosurgery unit, with subsequent hysteresis measurements using 30, 15, and 5 W. Each power step was performed using a measuring sequence of 25 min. One minute after stable baseline (~37.5°C) was established, power was applied for a duration of 1 min, followed by a cool-down period of 23 min. Ten samples were measured with the active electrode in position 1, 10 samples were measured with the active electrode in position 2, and 10 samples were measured with the active electrode in position 3. Measurements with an implant, without an implant, and with different active electrode positions were performed in arbitrary order.

The three hypotheses (H1, H2, and H3) were tested using linear mixed effects (LME) models. A full model was first used with the implant, power, position, and direction as fixed effects, and the tissue sample was used as a random effect, with a random intercept and slope for power, including all two-way interactions. Due to significant interactions between power and the implant, separate analyses were performed for each level of power using a model with the implant, position, and direction as fixed effects. To determine the effects of power on temperature increases, separate models were used with and without implants and using power, position, direction, and their two-way interactions as fixed effects.

*P* < 0.05 was considered significant after Bonferroni correction for multiple comparisons. Analyses were performed using the Statistics Toolbox in Matlab R2014b.

## Results

**Figure 4:**
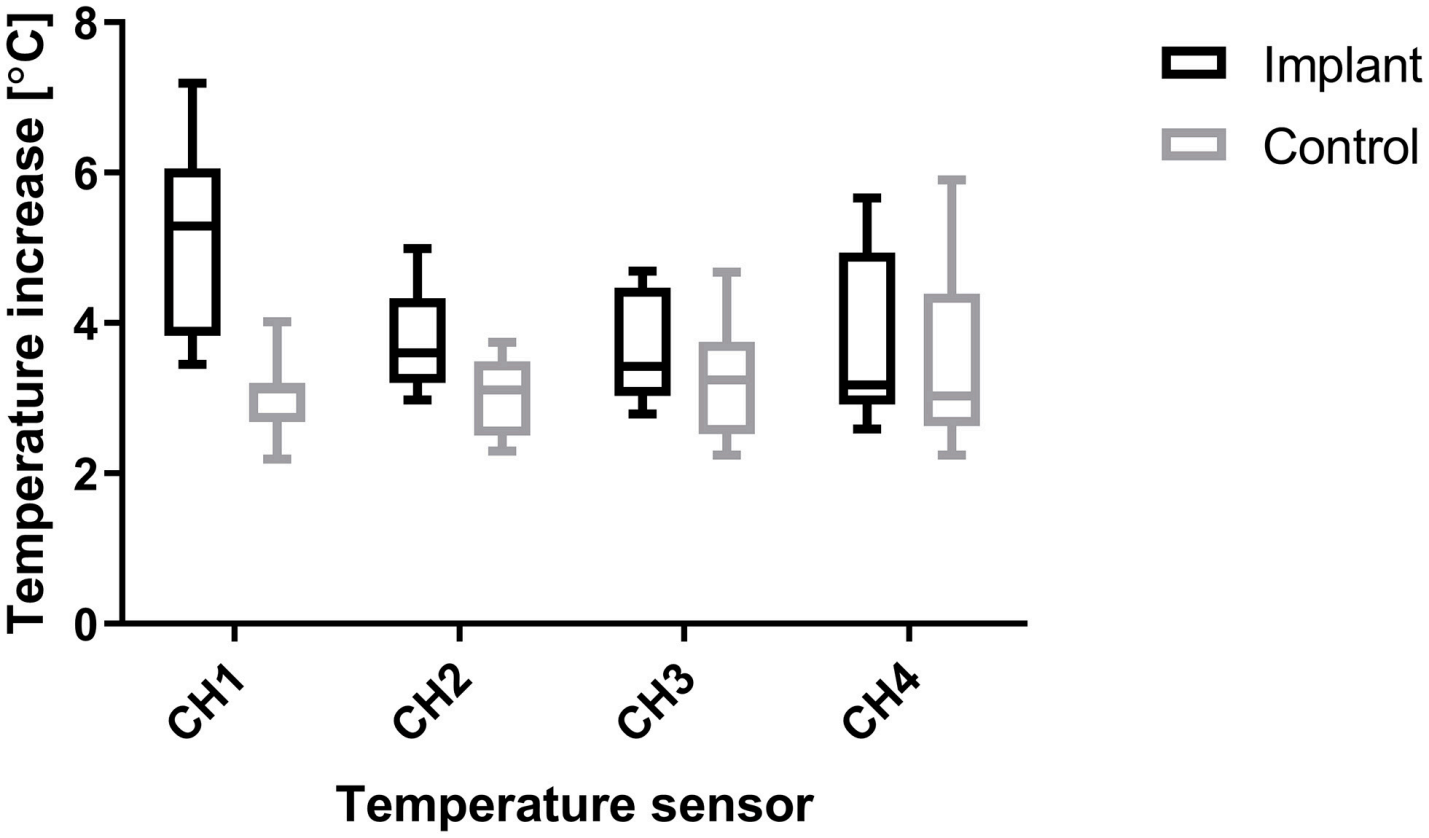
Distribution of temperature increases for sensor positions CH1 (2.5 mm from edge of implant) to CH4 (17.5 mm from edge of implant), pooled over all levels of distance and power, showing that the largest temperature increases were measured closest to the implant (CH1).

The tissue temperature at sensor position CH1 was most influenced by the existence of the implant (Figure 4). Therefore, we chose temperature measurements at sensor position CH1 to determine our results.

**Figure 5:**
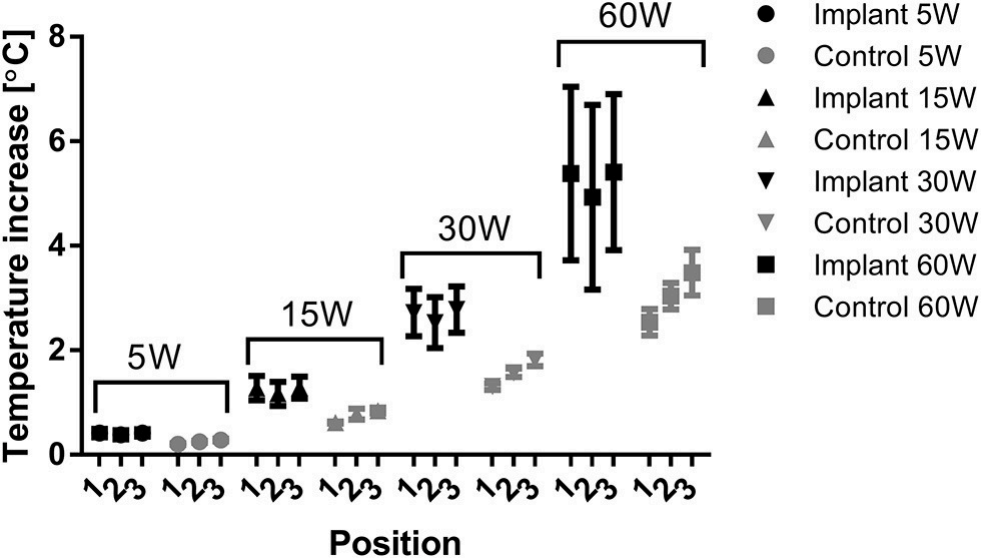
Temperature increase for tissue with and without implant according to power setting and active electrode position, represented as the means of each group with 95% confidence intervals of the means as error bars.

**Figure 6:**
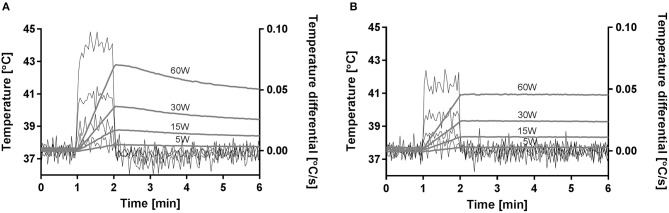
Mean temperature increase for tissue close to the metal implant **(A)** and for the control without an implant **(B)**.

For each power level, the temperature increase in the tissue with an implant was significantly higher than in the non-implant control tissue (Table 2). The active electrode position and the direction of the applied power change (hysteresis test) had no significant effects on temperature increase at any of the power levels. The applied power level had a significant effect on the temperature increase due to the implant (Implant vs. Control, Table 2), and higher power levels caused higher temperature increases both in tissue with an implant and in control tissue (Figure 5). With the implant in the tissue, the active electrode position had no significant effect on the relationship between power and temperature increase. In the control tissue, there was a significant interaction between the active electrode position and power (*p* < 0.001). As shown in Figure 5, the effect of the power setting on tissue heating increased from position 1 to position 3. In our model, the increases in temperature over time at power levels of 5, 15, 30, and 60 W were close to linear, as shown in Figure 6. The mean temperature increase per watt was 0.088°C/W during 1 min of applied electrosurgery (reaching approximately 43°C after 1 min of 60 W application). In some cases, the tissue temperature after 1 min at 60 W increased above 43°C.

**Table 2:** Factors influencing tissue heating during electrosurgery.

**Implant vs. control**	**Mean difference (°C/min)**	**95% CI (°C/min)**	**P (Bonferroni-adjusted)**
5 W	0.16	0.11–0.22	<0.001
15 W	0.50	0.33–0.66	<0.001
30 W	1.11	0.78–1.45	<0.001
60 W	2.22	1.56–2.89	<0.001
**Effect of power setting**	**Mean effect (°C/W/min)**	**95% CI (°C/W/min)**	**P (Bonferroni-adjusted)**
With implant^*^	0.088	0.078–0.099	<0.001
Control, position 1	0.042	0.039–0.046	<0.001
Control, position 2	0.051	0.048–0.054	<0.001
Control, position 3	0.059	0.054–0.064	<0.001

* *Different positions not included had no significant effects on the relationship between power and temperature increase*.

## Discussion

We have shown that a metal implant influences the electrosurgery current path and thereby the tissue temperature during monopolar electrosurgery. The temperature was higher with higher electrosurgery power. In our model, the temperature increased by ~0.09°C/W/min, which meant that the temperature could increase from a baseline temperature of 37.5°C to more than ~43°C after 1 min of 60 W application. However, regarding the electrosurgery active electrode distance from the implant, there was no significant linear association between distance and temperature increase. Based on the statistical results, H1 and H2 were accepted. However, H3 was rejected for tissue with an implant.

In the control group with no implant, there was a significant relationship between power and temperature increase, although it was lower in magnitude. We believe that this temperature increase was a function of the unmodified path of the electrosurgery current in the tissue. The influence of the implant on temperature was therefore presented as a comparison of the implant and control groups (Table 2).

Several variables influence the temperature increases in tissues with current passing in vicinity of a metal implant. However, will canalization of the current and increase in temperature reach the level of tissue damage? We may conclude that the conventional use of electrosurgery is performed with good safety margins when considering the risk of tissue-damaging temperatures. In this study, we analyzed the temperature increase after a 1-min electrosurgery activation period. In many cases, this application is discontinuous; however, some procedures may require continuous application for up to 1 min or more. In this study, we used an active electrode within a large cross-sectional area. Most normal electrosurgery procedures use a small contact area between the electrode and the tissue to achieve high current density. A small contact area provides high current density and makes it possible to heat the tissue in a controlled area; however, it also leads to higher resistance and, therefore, less current and less potential temperature increases around the implant. Due to the controlled situation in our model, we used an active electrode with a large surface area to avoid carbonization of the tissue at the active electrode. This electrode had much lower resistance and better contact with the tissue, thus differing from electrosurgery used in clinical practice. During the clinical use of electrosurgery, resistance between the active electrode and the tissue will fluctuate greatly, and most of the power will be distributed in the active electrode area. The way the surgeon handles the active electrode (e.g., activating time, tissue contact, open circuit activation, and amount of carbonized tissue) will also influence the current. Another variable that could influence the current going through an implant is the placement of the dispersive electrode. In our model, the implant was placed in the middle of the current path between the active and dispersive electrodes to enhance the reproducibility of the electrode setup.

Pettersen et al. (29) used Finite Element Model computer simulation to show that the points of maximum heating were outside the metal–tissue boundary, and found that heat passes from the tissue and into the metal implant ends, leading heat energy away from the tissue closest to the implant. Due to this cooling, the tissue closest to the metal was colder than the surrounding tissue around the implant ends. The temperature as a function of distance from the implant ends had rather sharp slopes, which in our model may explain large variations in the temperature probe placed closest to the implant. Pettersen et al. (29) also found that temperature “hot spots” are influenced by the size of the implant and the thermal capacity volume with high current density. An implant with more mass and higher thermal capacity will carry the heat away from the tissue close to the implant; therefore, the hottest area in the tissue will be slightly away from the implant. Therefore, it is possible that some areas had considerably larger temperature increases than those discovered by our closest temperature sensor (CH1). The present study was performed using power and distance between the active electrode and implant as variables. In the Pettersen et al. (29) study, authors theoretically showed that the length and shape of the implant influenced the temperature increase. With a longer implant, we can say that the current has more benefit taking the “shortcut” through the implant, and the temperature increase in the tissue at the ends will be higher than that found in the present study. Hence, we recommend that special precautions should be taken with regard to time and power and when long implants with sharp edges are present.

When implants (like hip bone implants or similar) are present during typical surgical procedures, we believe it is safe to use monopolar electrosurgery. However, the influence due to implant length and shape has to be considered. For example, a long scoliosis implant will have more influence on the current than a shorter implant. We could also expect that the size of the implant is influential; bigger implants and higher heat capacity keep the tissue at lower temperatures. The shape of the implant could also have a major influence on the temperature. For example, a long implant that ends in a sharp area or a screw could cause the current to be canalized to a small area and lead to higher current density and possible tissue damage. This could also occur with an active implant such as a DBS electrode with a long wire that ends in a small area in the brain. Therefore, it's advisable that the use of monopolar surgery in combination with DBS is contraindicated.

There was a certain weakness in our model because it was an *in vitro* model using non-vascularized tissue with no blood flow. The amount of blood flow in tissue is presumably an important variable that determines the degree of the temperature increase. Although activation times lead to increases in temperature, the blood flow in the tissue will counteract this effect. The blood flow will also influence the decrease in temperature during non-activation periods (pause) during electrosurgery. However, because the degree of vascularization depends on the tissue type, our model represents a worst case scenario with no vascularization.

In conclusion, temperature increases in tissue close to an implant are associated with passing monopolar electrosurgery current. Higher power leads to higher temperatures in tissue close to the implant. There was no linear relation between the active electrode distance from the implant and the temperature increase. This study clearly showed that electrosurgery current can be channelized through a metal implant. Furthermore, the resulting temperature increase is often within what can be considered safe limits. There is a significant risk of tissue damage when long activation times or high power levels are used, especially when long implants are present.

## Author Contributions

TM contributed to research design, protocol, research idea, development of experimental tools, conduction of experiments, main writer of manuscript, data analyses, and drawings. FP contributed to research design, protocol, development of experimental tools, writing of manuscript. HK, JH, and ØM contributed to research design, protocol, writing of manuscript. CT contributed to data analysis, research design, protocol, writing of manuscript. GK contributed to clinical interpretation, research design, writing of manuscript. AB contributed to development of experimental tools research design, protocol, writing of manuscript. SG contributed to research idea, research design, protocol, writer of manuscript. LF contributed to surgical clinical interpretation, research design, writing of manuscript.

### Conflict of Interest Statement

The authors declare that the research was conducted in the absence of any commercial or financial relationships that could be construed as a potential conflict of interest.
